# Theoretically Guided Iterative Design of the Sense2Quit App for Tobacco Cessation in Persons Living with HIV

**DOI:** 10.3390/ijerph20054219

**Published:** 2023-02-27

**Authors:** Rebecca Schnall, Paul Trujillo, Gabriella Alvarez, Claudia L. Michaels, Maeve Brin, Ming-Chun Huang, Huan Chen, Wenyao Xu, Patricia A. Cioe

**Affiliations:** 1 School of Nursing, Columbia University, New York, NY 10032, USA; 2 School of Public Health, Columbia University Mailman, New York, NY 10032, USA; 3 School of Engineering, Case Western Reserve University, Cleveland, OH 44106, USA; 4 Department of Computer Science & Engineering, University at Buffalo, The State University of New York, Buffalo, NY 14260, USA; 5 School of Public Health, Brown University, Providence, RI 02903, USA

**Keywords:** mHealth, tobacco cessation, HIV, Design Sessions, focus group sessions, qualitative

## Abstract

The use of mobile health (mHealth technology) can be an effective intervention when considering chronic illnesses. Qualitative research methods were used to identify specific content and features for a mobile app for smoking cessation amongst people living with HIV (PWH). We conducted five focus group sessions followed by two Design Sessions with PWH who were or are currently chronic cigarette smokers. The first five groups focused on the perceived barriers and facilitators to smoking cessation amongst PWH. The two Design Sessions leveraged the findings from the focus group sessions and were used to determine the optimal features and user interface of a mobile app to support smoking cessation amongst PWH. Thematic analysis was conducted using the Health Belief Model and Fogg’s Functional Triad. Seven themes emerged from our focus group sessions: history of smoking, triggers, consequences of quitting smoking, motivation to quit, messages to help quit, quitting strategies, and mental health-related challenges. Functional details of the app were identified during the Design Sessions and used to build a functional prototype.

## 1. Introduction

Of the approximately one million persons living with HIV (PWH) in the United States (U.S.) [1], it is estimated that approximately 50% smoke cigarettes [2], approximately four times the prevalence (12.5%) observed in the general U.S. adult population [3]. Consequently, PWH experience substantial tobacco-related morbidity and mortality. In PWH, after achieving and maintaining a suppressed viral load, smoking cessation is the next most important health behavior to maximize both quality of life and life expectancy. Indeed, PWH who quit smoking upon entering HIV care gain greater than 5 years of life expectancy as compared to those who enter HIV care and continue smoking [4,5].

Given the few randomized controlled trials (RCTs) examining smoking cessation interventions for PWH and the major methodological limitations of many of these studies (e.g., lack of randomization, comparison conditions, treatment fidelity assessments, and abstinence verification tests), it is critical to develop evidence-based tobacco cessation interventions to address the complex and unique needs of PWH (e.g., risk factors and treatment needs) [6,7]. Tailored cessation interventions for PWH need to focus on the factors specific to PWH which are associated to date with poor success with tobacco cessation efforts. For example, neurocognitive deficits are common among PWH and are associated with poor quit rates and relapse, supporting the need for unique interventions for PWH [8].

**Figure 1:**
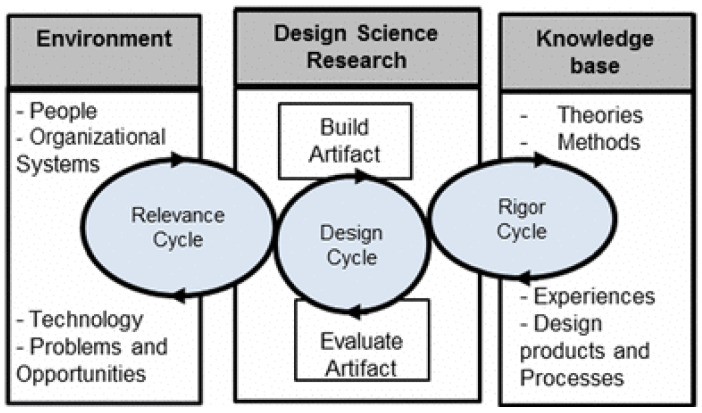
Information System Research (ISR) framework for design of mHealth technology adopted from [17].

In recent years, the use of mobile health (mHealth) technologies for tobacco cessation has gained popularity, such as Text2Quit [9] and the National Cancer Institute’s (NCI’s) SmokeFree Text [10]. While these programs have demonstrated positive effects in some populations [11] their functionality is limited because text messaging is in a pre-defined alert setting, and cannot timely respond when assistance to resist cravings is needed, or when relapses occur [12] To address this need, our study team developed a closed-loop solution that integrates both behavioral assessment (using wearable sensors) and a just-in-time cessation intervention for smokers [13]. Specifically, our Sense2Quit App builds on the extant evidence that text messaging can be an effective tool for improving smoking cessation in the general population and addresses specific challenges to tobacco cessation for PWH: slips, relapses, and difficulties associated with neurocognitive deficits and supports self-efficacy [14]. Sense2Quit is a multi-component intervention that links a smartphone app to a smartwatch and provides just-in-time quit reminders, in order to curtail relapses and avoid potential triggers. The artificial intelligence algorithm [15] was developed using two arm bands to interpret time-series wrist-worn motion sensor data to detect smoking gestures and motions—a highly accurate technology that differentiates “lighting up” from other similar motions. The Sense2Quit App will leverage this artificial intelligence algorithm and use a smartwatch to detect when and where smoking activity occurs so that an intervention message can be sent in real time to the participant via a linked smartphone app to help prevent the slip (a puff or two) from becoming a full relapse. Real-time feedback is critical, especially in the first few weeks of a quit attempt [16], to understand a smoker’s daily routine and physical activities. The purpose of this paper is to report on the first stages of the design and development of the Sense2Quit App. The design of the Sense2Quit App was guided by the Information System Research (ISR) framework (Figure 1) [17]. Three cycles comprise this framework: Relevance, Rigor and Design. In the Relevance Cycle, we use focus group methodology to identify the needs of the end-users. In the Rigor Cycle, we reflect on the extant technologies, theories and other tools which have been developed to improve tobacco cessation. In the Design Cycle, the focus is on designing the user interface and critically evaluating the usability of the technology. This process has been well established for use for the design of mHealth tools [18].

This paper focuses on the Relevance and Design Cycle/Build Artifacts & Processes. The process for Design Cycle/Evaluate for the Sense2Quit App is described elsewhere [19].

## 2. Materials and Methods

### 2.1. Study Design

This study described the use of a qualitative observational study to inform the design of a mobile app to help people living with HIV (PWH) in their smoking cessation efforts.

### 2.2. Recruitment and Eligibility

This study was comprised of 27 participants across seven different focus group sessions: five Relevance Groups (n = 22) and two Design Sessions (n = 5). There were between 2 and 8 participants in each Relevance Group. The same participants participated in both Design Sessions with the exception of one participant who only attended the first session. None of the participants in the Relevance Groups participated in the Design Sessions. We used a convenience sampling approach for the recruitment of participants. Data collection continued until saturation of data was reached. Inclusion criteria were limited to: (1) being over the age of 18, (2) HIV and/or AIDS diagnosis, (3) able to communicate and read in English, (4) comfortable taking a survey on a tablet/iPad, and (5) a current or former smoker (‘smoker’ being defined as smoking 5 or more cigarettes per day). The focus group sessions took place from December 2021 through February 2022. All focus groups took place in person in New York City, New York.

### 2.3. Procedures

The Institutional Review Board of Columbia University Medical Center reviewed and approved all research activities. Prior to each focus group discussion, a staff member provided study participants with an explanation of the study procedures and outlined details of the consent form. All participants completed and signed a consent form before participating in study activities. We employed a semi-structured focus group guide using open-ended questions and probing questions. Focus group and Design Session questions are listed in Table 1. The moderator facilitated the focus group sessions, and participants were encouraged to discuss their thoughts on perceived barriers and benefits of smoking cessation. All focus group sessions were audio-recorded. Enrollment continued until saturation of themes was reached. Participants were compensated USD 40 for their time.

### 2.4. Data Analysis

Audio recordings from all focus groups were transcribed. Transcripts were uploaded and analyzed using Dedoose^®^ Verion 9, analytical software. Two thematic frameworks guided the data analytic plan—the Health Belief Model [20] and Fogg’s Functional Role Triad [21]—and guided the code development, which was incorporated into the Dedoose software. The Health Belief Model [20] serves as an example of the value-expectancy theory, and posits that a person’s desire to get well or avoid illness completely propels people to avoid behaviors [20]. Fogg’s Functional Triad addresses a “functional” view of persuasive computers, and reference computers as interactive technology to facilitate behavior change that has the ability to shift or alter a person’s attitudes or behaviors [15,21]. Perceived barriers and benefits were the two key constructs taken from the Health Belief Model which guided our analysis. Two research team members reviewed the transcripts and generated a set of codes through inductive content analysis. The final codebook for both sets of focus groups was uploaded to Dedoose^®^ for storage. Thematic analysis of the focus group sessions investigated the salient and recurring themes across all sessions. Quotations were assigned one or more codes if they fit the definitive criteria of such codes from the established codebook. Coding for each transcript was deemed saturated when no new information could be coded based on the codebook [15].

Findings from the Relevance Cycle/Focus Group sessions were used to generate categories for the app prototype: Notifications, Customization, Games, Savings Tracker, and Chat Function. Next, Fogg’s Functional Role Triad was used as a framework to analyze the data gathered during the Design Sessions. Fogg’s Functional Role Triad proposes that health behaviors can be effectively influenced through technological mechanisms that utilize three constructs: Medium, Social Actor, and Tool [21].This framework has been used in previous studies to guide the design of mHealth tools for improving health outcomes in PWH [22]. Mediums refer to the various modes through which information is disseminated and experiences are contained in the technological platform [21]. Social Actor refers to the force that creates a relationship with the user by modeling appropriate health behavior, rewarding appropriate health behavior, or providing social support [21]. Tool refers to a technological function that increases a user’s capacity to achieve a desired health behavior [21]. Coding discrepancies were discussed until a consensus was reached.

## 3. Results

### 3.1. Study Sample

Fifteen participants identified as male at birth and the remaining identified as female at birth. Our study participants ages ranged from 30 to 76 years old, with a mean age of 54 years. Nearly all of our study participants were from racial/ethnic minority groups. A total of 22 of our study participants self-identified as Black/African American, 4 as other race (non-White), 1 as Asian American and 1 as White. Eight study participants self-identified their ethnicity as Latino.

### 3.2. Focus Group Findings

Seven major themes emerged from our focus group session: history of smoking, triggers, consequences of quitting smoking, motivation to quit, messages to help quit, quitting strategies, and mental health-related challenges. Each theme was then further coded as a perceived barrier or perceived benefit. Sample quotes to illustrate each of them and sub-code are listed in Table 2.

**History of smoking.** Many respondents noted that they had a history of chronic cigarette smoking over multiple years. Several respondents (coded 5 times) noted that they wanted to quit smoking due to their long history of cigarette use.

**Triggers.** Many respondents noted specific triggers that influenced them to smoke on a daily basis (coded 96 times). Of the respondents who reported triggers, the majority (coded 46 times) stated that eating food and drinking coffee prompted them to smoke. Many respondents (coded 31 times) attributed their trigger to smoke with drinking alcohol. Some respondents (coded 6 times) claimed that their current or previous drug use was a trigger for smoking.

**Consequences of quitting smoking.** Several respondents noted the unintentional consequences of smoking cessation as perceived barriers (coded 15 times). Of these respondents, many stated that weight gain was a perceived concern of smoking cessation (coded 6 times). Some of the respondents noted turning to marijuana use as a consequence of quitting cigarettes (coded 4 times).

**Motivation to Quit.** Many respondents acknowledged health as a driver to quit smoking (coded 47 times). Of these respondents, most (coded 31 times) wanted to quit or had previously quit due to personal health concerns. The remaining respondents (coded 16 times) wanted to quit due to loss of a loved one from smoking-related health complications. Other respondents stated that they wanted to quit for some reason unrelated to their health (coded 22 times). Of these respondents, most (coded 14 times) claimed that the financial savings from not buying cigarettes would entice them to quit. A few participants (coded 3 times) noted that the smell of cigarettes would motivate them to quit.

**Messages to Help Quit.** Several respondents who recalled commercials (coded 23 times) felt that negative imagery would make them so uncomfortable that they would instead want to smoke more. All respondents were asked to provide messages that would prompt someone to quit or stop smoking (coded 181 times). Most of the respondents (coded 55 times) recalled commercials with negative imagery that made them want to quit. Others (coded 47 times) shared that positive reinforcement would work best for them. Some respondents (coded 32 times) felt that offering smoking substitutes would prompt them to not smoke.

**Quitting Strategies.** Many respondents offered strategies for smoking cessation (coded 39 times). Most respondents (coded 22 times) stated that having another person offer social support would help with the quitting process. Some respondents (coded 9 times) thought that a step-wise process could help people gradually quit.

**Mental Health-Related Challenges.** Many respondents shared that smoking is or previously was a coping mechanism for their mental health concerns (coded 31 times).

Design Session findings were organized by Fogg’s Functional Triad, which has been successfully used as an approach in previous research to guide the development of mHealth tools [21]. Findings were coded as a Social Actor, Tool—Imagery, Tool—Customization and Medium. Sample quotes from the Design Sessions can be found in Table 3.

*Social Actor*. Some respondents noted their concern over discussing their quitting process with other people, specifically in the context of a group discussion (coded 12 times). Many respondents stated that having someone to keep them accountable would improve their quitting process (coded 43 times). Of these respondents, the majority (coded 32 times) felt that connecting with someone with personal experience quitting smoking would be most effective. Several respondents (coded 11 times) stated that conversing with a live person in a chatroom would encourage their quitting process.

*Tool—Imagery*. Some respondents felt that images reflecting the negative impacts of smoking would deter them from using the app to quit smoking (coded 3 times). Many respondents said that using bright and “fresh” colors in the app design would make them want to use it more (coded 28 times). Several respondents mentioned that including images of their loved ones would motivate them to quit smoking (coded 10 times). Several respondents said that including images of people living healthy lifestyles would make them want to use it more (coded 8 times). Some respondents said that using images, rather than numbers would make them more likely to use the financial tracker component (coded 4 times).

*Tool—Customization*. Many respondents stated that having the option to customize their notifications would help in the quitting process (coded 19 times). Several respondents stated that having the option to customize their financial tracker would help in the quitting process (coded 9 times).

*Medium*. Some respondents stated that receiving notifications too frequently in an app would deter them from using the system (coded 4 times). Several respondents agreed that a defined chat function would work best in a group format (coded 13 times). Many respondents stated that a financial tracking component would improve their quitting process (coded 23 times) and many agreed that a game component would keep them distracted from wanting to smoke (coded 25 times). Several respondents stated that receiving notifications from an app would help them quit smoking (coded 17 times).

Many respondents stated that having access to accurate health information related to smoking would benefit their quitting process (coded 27 times). Some respondents stated that they would benefit from an app that connected users to people who can relay information directly (coded 4 times).

## 4. Discussion

Similar to findings in previous studies of smokers in the general population and in PWH, findings from this study suggest that most people struggled to quit because they used smoking as a coping mechanism for various daily stressors, mental illness, and concurrent drug use [23,24,25,26]. Additional concerns around smoking cessation included weight gain, adjustments to routines/daily habits of smoking, as well as social pressures. Considering this, participants shared that the most significant motivators to smoking cessation would be awareness of serious illnesses and morbidities due to smoking habits and learning about other ways to handle stress, illness, and drug use. Loss-frame messages [27] that were described as useful were related to the increased risk of illnesses such as cancer, cardiovascular disease, or emphysema, as well as those referencing loved ones and second-hand smoke for children.

Participants discussed which types of messages would be most effective in changing their behavior and reflected on similar findings in another study in PWH [28]. Specifically, they mentioned that gain-framed messages [27] would be more useful than loss-framed messages. One participant noted that they would like a positive alert such as “You can do it!”, with most study participants in agreement that they would like to somehow be reminded of their quit plan at least once a day. On the other hand, some participants noted that they do not appreciate a “scare tactic” [29] within the application.

Timing of messages was also an important consideration, which has been identified as a facilitator in previous studies on mHealth engagement. For instance, participants discussed that after mealtimes (at least three times a day) as well as before and after bed would be useful timing for smoking cessation reminders. On the other hand, participants explained that hourly alerts would be too much. Further, there is the opportunity to link smoking cessation with adherence to antiretroviral medications for PWH, particularly those persons who are struggling with both of these health challenges.

Positive reinforcement was highly desired, with a specific preference for being reminded of accomplishments, rather than viewing ‘negative’ notifications in reference to one’s quitting journey. The need for such messages arises from specific triggers, with most participants in agreement that urges around mealtimes, morning alarms, and generalized stress could be effectively subdued. This feedback reveals a need for customization within app functioning in that it should be tailored to a participants’ needs, focused on common daily triggers, incentivize cessation behaviors and linked to external resources. Additionally, many participants noted a preference for a responsive element (i.e., someone to talk to or respond) in the app which would further enhance the functionality of the app.

Through a user-centered participatory design, key features for improving the acceptability of this mHealth tool were addressed. In the past, few mHealth tools for smoking cessation have incorporated HIV-specific content. Therefore, this user-centered design approach, which elicits information directly from PWH, has the potential to improve tobacco cessation in PWH.

### Limitations

Due to demographic limitations, the research study population was limited to those in the greater New York City (NYC) area. Nonetheless, due to the high prevalence of PWH (approximately 140,000 total in 2021), especially those in both lower- and middle- class [30], these findings show promise in improving mHealth research. Furthermore, participants were recruited based on interest and were thus likely to have an affinity for use of technology. Nonetheless, a significant strength of this study is the recruitment of a racially diverse sample, which is often not achieved in research studies.

## 5. Conclusions

Mobile health technology can be leveraged to manage chronic illnesses and help change health behaviors. Principal features that were identified included encouraging messages, reminders and links to external resources. Information collected during the Design Sessions will be useful for refining the user interface of the app.

## Figures and Tables

**Table 1:** Focus Group Guides.

Relevance Focus Groups
What are the barriers to quitting smoking? What messages would motivate you to not light up your cigarette? What information would you like to see included in an app? What messages would deter you if were about to start smoking a cigarette? How frequently do you want to receive reminders about your quit plan? How frequently do you want alerts about your accomplishments? What are your triggers? And what is the best time for you to receive an alert about the trigger?
Design Session 1.0: Optimal Features
Starter: If you had an app to help you quit smoking, what messages would stop you from smoking a cigarette? *(Referring to slides/handout) [see Supplementary File S1]* Imagine each of the broad categories as separate screens on a mobile app. What first comes to mind when you think of this category? a. How does the category (e.g., content, features, functions) relate or do not relate to your everyday experiences? b. What challenges can you expect in using the features and functions in this category? What specific types of content and features you think should be included in this category of the mobile app? a. For each category, please draw a sketch of what you would imagine it to look like in the mobile app. b. How could this category be shown differently? What additional information would you like to see from a mobile app related to smoking cessation? a. What additional categories would you like to see? b. If applicable, what’s your experience using other kinds of health apps (apps to improve your health)? i. Did you like using them? Why or why not?
Design Session 2.0: Interface
Starter: If you have used an app to improve or monitor your health, what did it look like? If you have not used such an app, what would you expect it to look like? *(Referring to slides/handout) [see Supplementary File S1]* Imagine each of the broad categories as separate screens on a mobile app. What images first come to mind when you think of this category? Please draw or describe these images. What specific types of content do you think should be included in this category of the mobile app (i.e., pictures, buttons, sounds, or movements from your phone)? Please draw or describe these specific features. For each category, please draw a detailed sketch of what you would imagine it to look like in the mobile app. Include the specific types of content that you have previously described. a. How does this compare this to the current prototype designs shown on the screen? If this was a separate page of the app, how would you get there from the home screen? *(Not referring to slides/handout)* How would you like the home screen to look when you first open the app? Please draw an example of what this should look like. What would make it easy for you to quit smoking using this mobile app? What additional information would you like to see from a mobile app related to smoking cessation? a. Where would you like to see additional resources to help you quit smoking?

**Table 2:** Findings from the Focus Group Sessions.

Code	Excerpts	
	Perceived Barriers	Perceived Benefits
History of smoking (n = 19)	*“My barrier is that I have…it’s like the routine that you get when you start smoking…when you’ve been smoking for a long time.*”—FG2 Participant *“I quit smoking for two years because I had a heart attack, which was from the cigarettes. Last year I started picking it up again.”*—FG1 Participant *“I started smoking three years ago because I was a part of the in-crowd. Everybody else was doing it, so let me go ahead and try it.”*—FG2 Participant *“Yes, the urges get so bad and like I really want to stop so bad, but I’ve been doing it for so long now, you know so it’s really hard. I’ve been smoking over twenty years, but you know I just.”*—FG4 Participant	*“I caught a bad asthma attack and I had to go to the hospital, because I was smoking cigarettes back-to-back. So, I had to go to the hospital, and I was like, you know what? Something’s got to give. Girl, you’ve got to get it together. Like, for real, for real. Do you want to stay alive, or you want to be somewhere else?”*—FG3 Participant *“What I went through then was horrible. Even my family was scared I was going to die. If you put the HIV with the smoking and you see how I was then, I was like, nah.”*—FG2 Participant
Triggers (n = 96)	*“For me, I get up in the morning, have coffee, I have to have a cigarette. After I eat, I have to have a cigarette. Certain things like that. It’s not just the nicotine, it’s to have something in your mouth; in between your fingers. That’s one of the barriers that I have.”*—FG2 Participant *“Well, I love to eat, and so you know. But after I eat, I want a cigarette.”*—FG4 Participant *“For me, I get up in the morning, have coffee, I have to have a cigarette. After I eat, I have to have a cigarette. Certain things like that.”*—FG2 Participant *“My barriers, number one, starts with my drinking…the alcohol. That’s where it all starts.”*—FG2 Participant *“Now, for me when I was doing drugs, I had to have cigarettes. When I drink, yeah, I had to have cigarettes, you know.”*—FG3 Participant	No quotes
Consequences of Quitting (n = 15)	*“There’s the good and the bad. For some people you don’t want to smoke cigarettes, eat a piece of candy or something. But then, you get diabetes.”*—FG1 Participant *“I am just worried about massive weight gain and stuff. That’s what I worry about.”*—FG1 Participant *“I started smoking, marijuana, because not smoking those cigarettes now. So now I’m smoking marijuana, but I’m smoking more marijuana…”*—FG3 Participant	No quotes
Motivation to Quit (n = 69)	No quotes	*“Ok, I know I smoked, how many pack of cigarettes day, right? You put that $10.00 up in the bank. And you have that other $10.00. And you look at yourself, you’d be like, wow, I saved all of this.”*—FG3 Participant *“Financially, it’s hurting me, but I am still spending that kind of money. That’s what I am saying. All that money I could be saving towards something else.”*—FG2 Participant *“I don’t smoke the whole day, but I will have a spray or something, because I don’t like to smell the smoke on me.”*—FG3 Participant
Messages to Help Quit (n = 181)	*“I turn around with the commercials because I hate the commercials. Every time I see a commercial that’s when I smoke because they piss me off…really, really piss me off…those commercials.”*—FG2 Participant *“The commercials just punish you. They don’t reward you. A lot of people are more receptive from rewards than punishment.”*—FG2 Participant *“They done had one person with their fingers falling off, I was still smoking.”*—FG3 Participant	*“For me, I saw cancer. I see those commercials on TV with the guy with the thing is his throat. That’s really scary.”*—FG2 Participant *“By see past people laying in the hospital bed with cancer or something like that. That would scare me.”*—FG2 Participant *“And it could just say to you, congratulations, Mr. Such and Such, you completed. You know and then the party confetti. And then a little turtle gets up and do his little dance.”*—FG #4 *“Or maybe something like that comes on and says, you know find something else to do. You know do some exercising.”*—FG3 Participant
Quitting Strategies (n = 39)	No quotes	*“You have to have somebody to check you, for real. That’s the only way. They keep bugging you until you stop.”*—FG1 Participant *“Tell me, ok, if you do this, I’m going to work with you on this. Or I’m going to help you with this. Or, matter of fact, I will go with you to the meeting; how about that?”*—FG3 Participant *“I’ll smoke a half a cigarette and then leave the rest and smoke the other half later, which helps me to cut down a lot.”*—FG1 Participant
Mental Health (n = 31)	*“You wake up and try to have a positive outlook, and then something just brings your day down…I need a cigarette.”*—FG1 Participant *“Mental health issues…if you’re going through like anxiety, depression. Probably loneliness. You need something to cope. So, I would say that is one, too.”*—FG3 Participant *“Yeah, stress is the number one thing, that you start smoking. Because if you can’t get nobody on the phone, like stressed out, and you normally…go to, but like my mom (inaudible) person. I said you know, since she’s gone now, right? I go to a cigarette.”*—FG5 Participant	No quotes

**Table 3:** Results from Design Sessions.

Code	Excerpts	
	Perceived Barriers	Perceived Benefits
Social actor (n = 55)	*“I don’t think we should share information.*”—DS2 Participant *“I might be jumping the gun, but you need a moderator because people like to go there when they got nothing to do with the group…”*—DS2 Participant *“Because you’ve always got a couple of knuckleheads that’s going to want to, you know. Because a lot of these groups, I’ve noticed that—especially Facebook groups, the person that—they have a moderator. Everyone just can’t get on and say anything they want. So, a moderator would be helpful.”*—DS2 Participant	*“Like he said, someone in the same…identifying with you. That’s what I was saying; somebody giving you real information and based on their experience or something. That would be great.”*—DS1 Participant *“You know, to look at. Somebody that’s maybe a success story. Hi. I’ve been smoke-free for something like that. Just a greeting. And you can meet some of my peers that have also been clean for a few minutes. And we can share some, you know, information and get you started.”*—DS2 Participant *“The apps need to have a place where you could maybe look for sponsors, if they been thinking about sponsors.”*—DS2 Participant *“If I am in a chat room, I would like to see you or at least see a picture of you to know who I am talking to…or a video would be ultimate.”*—DS1 Participant *“I’d rather live, you know, because that shows a lot of your expressions and, you know, when you’re saying something to me.”*—DS2 Participant
Tool: Imagery (n = 68)	*“That negative stuff…I don’t want to see that lady with that hole talking. That was terrible.”*—DS1 Participant	*“Yeah. I think colors are important. Certain colors kind of put my mood in different ways. Like she was saying, it’s the sky and sky blue. You know, I like those type of colors. They seem to make people’s attitudes a little milder than some other colors.”*—DS2 Participant *“I would say something maybe like sky color. Like when I’m doing something outdoors.”*—DS2 Participant *“They want to quit smoking. So, they want something bright and cheery.”*—DS2 Participant *“I would say my kids because I don’t smoke around them.”*—DS1 Participant *“Positive message sent…something like that. Like, they’d send something positive to me…positive message…you can do it; send you pictures of someone how has stopped smoking.”*—DS1 Participant *“Showing the benefits to my health. That would help. Showing positive pitches.”*—DS1 Participant *“Healthy person with a big heart beating blood.”*—DS2 Participant *“What’s you’re saving on and the bank is getting bigger and bigger. You actually see the money grow.”*—DS1 Participant *“I like the piggy bank. Yeah. You saved all that.”*—DS2 Participant
Tool: Customization (n = 28)	No quotes	*“I am leaning a little more towards my own message because I only know what excites me. Nobody knows what really gets me going deep down for me…all of us. A lot of things that affect us we don’t bring up for a myriad of reasons…embarrassment; forgetfulness. If I can customize it and I know I am going to be the only one that sees this.”*—DS1 Participant *“Yeah. So, you would be able to personalize the notifications. Like for me, it would be a picture of my niece, my girlfriend, if I had one.”*—DS2 Participant *“Personalization, yeah. Customizing it to your, you know, your age group and your culture and your area…”*—DS2 Participant *“I put down here show what I can buy when I stop. If you stop for ten years, you can buy a car. If you stop for five years, you can buy a dress…if you stop a month, you can buy this new dress. Actually, show the price of the cigarette against the price of the dress…wow, if I stop for that amount of time, I can get those shoes. Peaked me a little bit.”*—DS1 Participant
Medium (n = 81)	*“(In other apps) I get notifications I don’t want”*—DS1 Participant *“I really don’t have much to say on that, only because all of these notifications are annoying the hell out of me now.”*—DS2 Participant	*“I think that’s (group chat) a good idea for an app, you know, that you can actually bring someone else in.”*—DS2 Participant *“And it gives me a goal…I saved $20 this week; maybe I could do $30 next week. Just something to keep you…that would interest me a little, probably…something to do. Maybe I could save a couple more dollars this week.”*—DS1 Participant *“Like the educational game. You answer the questions correctly, you get to go to next level. It’s pretty cool. I would do that. If you answer the questions correctly, you go on to the next level. If you keep asking the questions correctly, you’ll get to the end eventually and win.”*—DS1 Participant *“Games in general that you could pick—because I know some people, just having a game would be enough, any game. Some people, just to take they mind off it. I’m kind of like that.”*—DS2 Participant *“I like what you said about a bell that notifies you. Maybe they have meetings…let you know when there is a meeting. Notification letting you know there is a meeting, like a smoke cessation meeting.”*—DS1 Participant *“Yeah. I’d like the notifications to come through because you know, I don’t go to—I’m not like that—I’m not going through my phone to see the dating site, get my emails, my (inaudible). That is why I do like to hear the ping.”*—DS2 Participant
		*“As I’ve been saying before, I think education is important. The more we know about HIV, the more we know about smoking, and I think that would be helpful. I am stealing your idea. I’ve got medical facts, but you write education…more than medical facts, it’s education about a lot of things.”*—DS1 Participant *“I’ve got to say, I’m going there looking for something to help me stop smoking, so, just the benefits and I can click on that and see what the benefits are.”*—DS2 Participant *“I would like an app that would help point you to information; people to talk to; that would have a list of people you can call and who would be helpful and talking you down from a cigarette, maybe.”*—DS1 Participant

## Data Availability

The data presented in this study are available on request from the corresponding author. The data are not publicly available due to privacy protection of participants.

## Supplementary Materials

The following supporting information can be downloaded at: https://www.mdpi.com/article/10.3390/ijerph20054219/s1, File S1: Sense2Quit.
