# Supplementary material for: Theoretically Guided Iterative Design of the Sense2Quit App for Tobacco Cessation in Persons Living with HIV
Source: Int J Environ Res Public Health. 2023 Feb 27;20(5):4219. doi: 10.3390/ijerph20054219 (PMC10001855; doi:10.3390/ijerph20054219)
Supplement: Supplementary file 1 [file ijerph-20-04219-s001.zip › ijerph-2223031-supplementary.pdf]

Sense2Quit

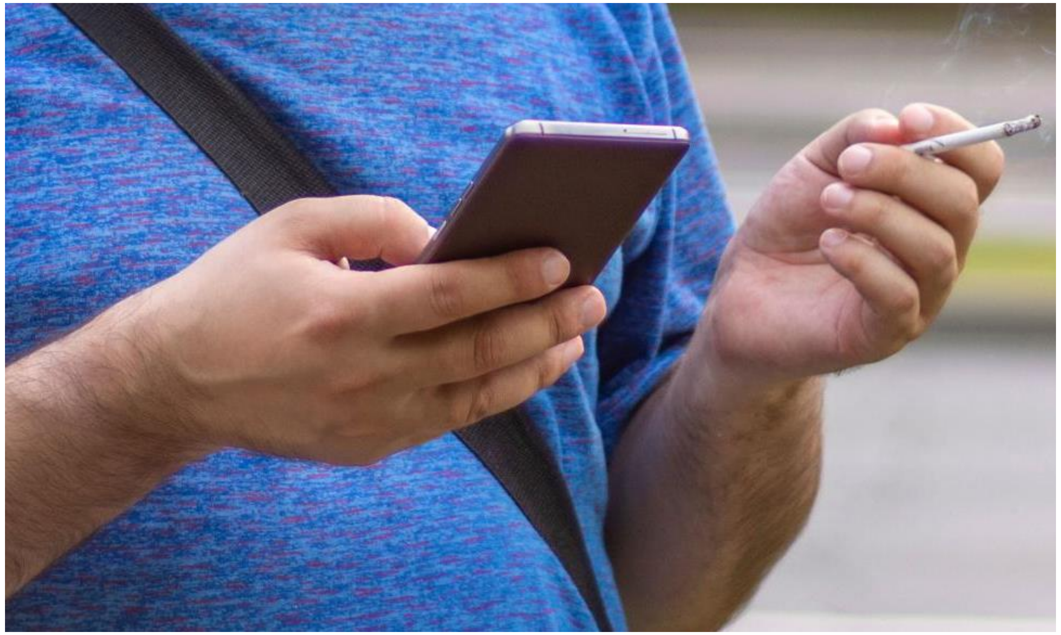

APP DESIGN SESSION 1.0

COLUMBIA
SCHOOL OF NURSING

Overview

Purpose: To create a highly usable and relevant mobile app for people living with HIV (PLWH) who smoke and want to quit smoking

2 Design Sessions: 1.0 (Optimal Features) and 2.0 (Interface)

- Today is Design Session 1.0

Goal 1.0: Identify features for the mobile app based on the information provided by earlier focus groups

- Content, features, and functions

If you had an app to help you quit smoking,

What messages would stop you from smoking a cigarette?

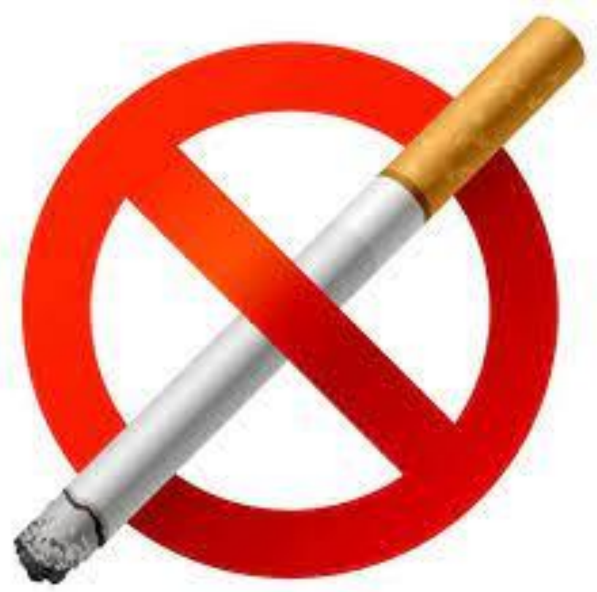

Please refer to your handout for the next 5 categories

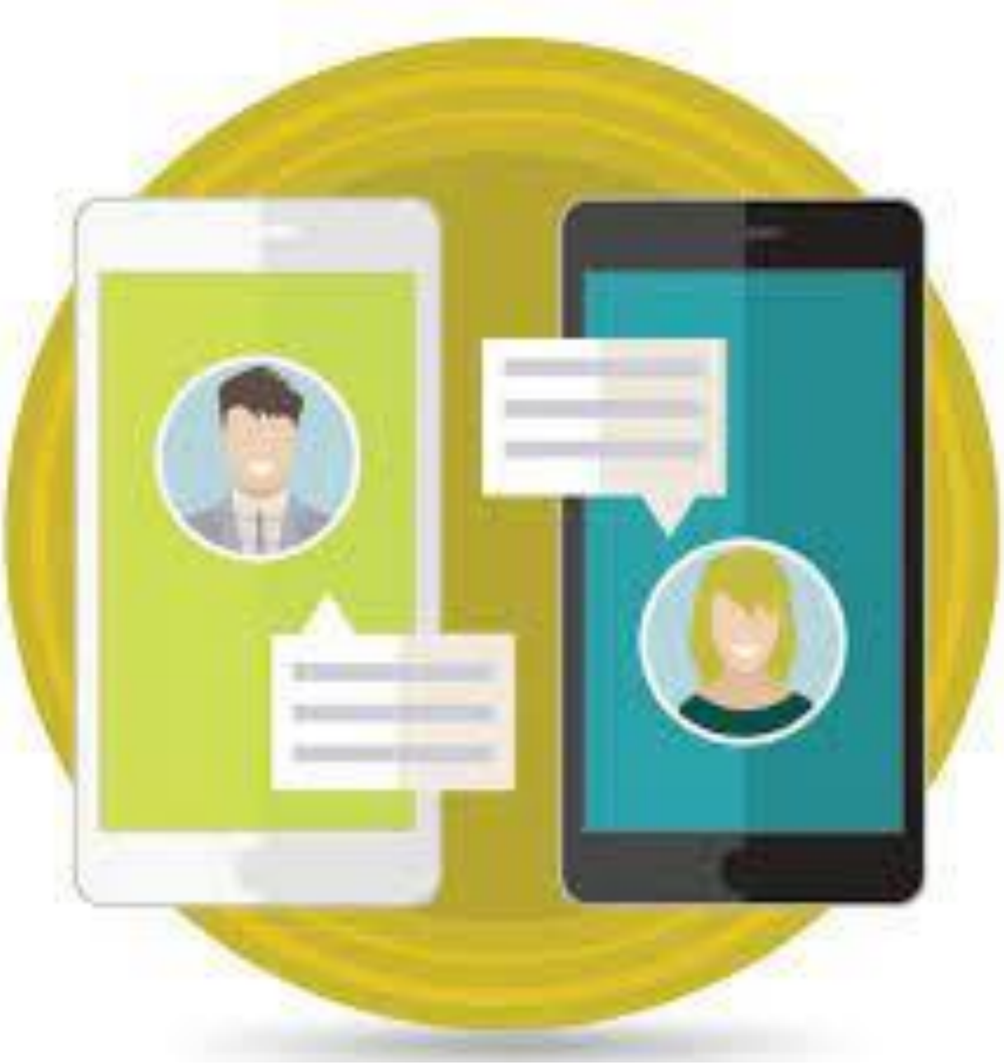

Category #1: Chat Function

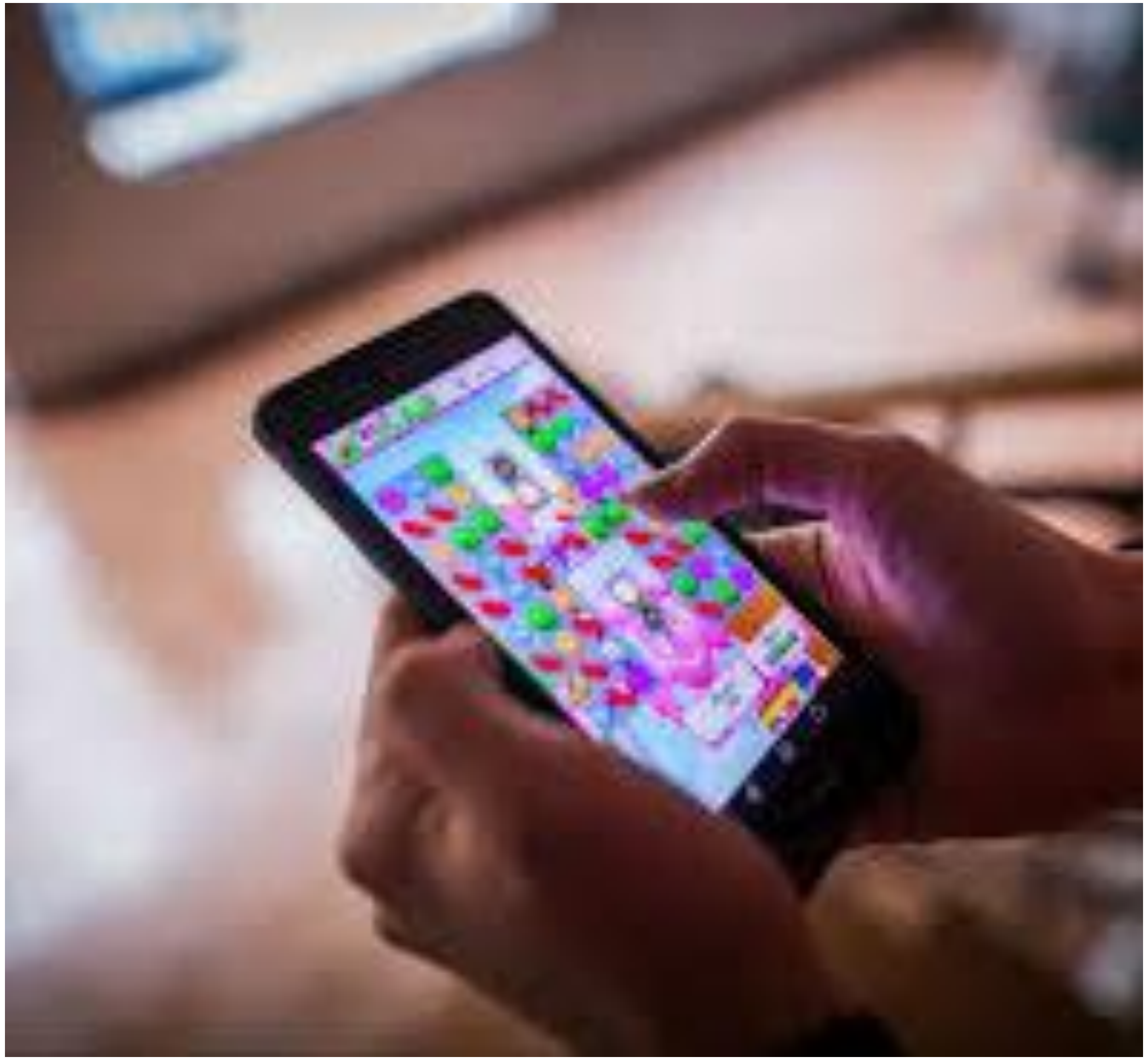

Category #2: Games

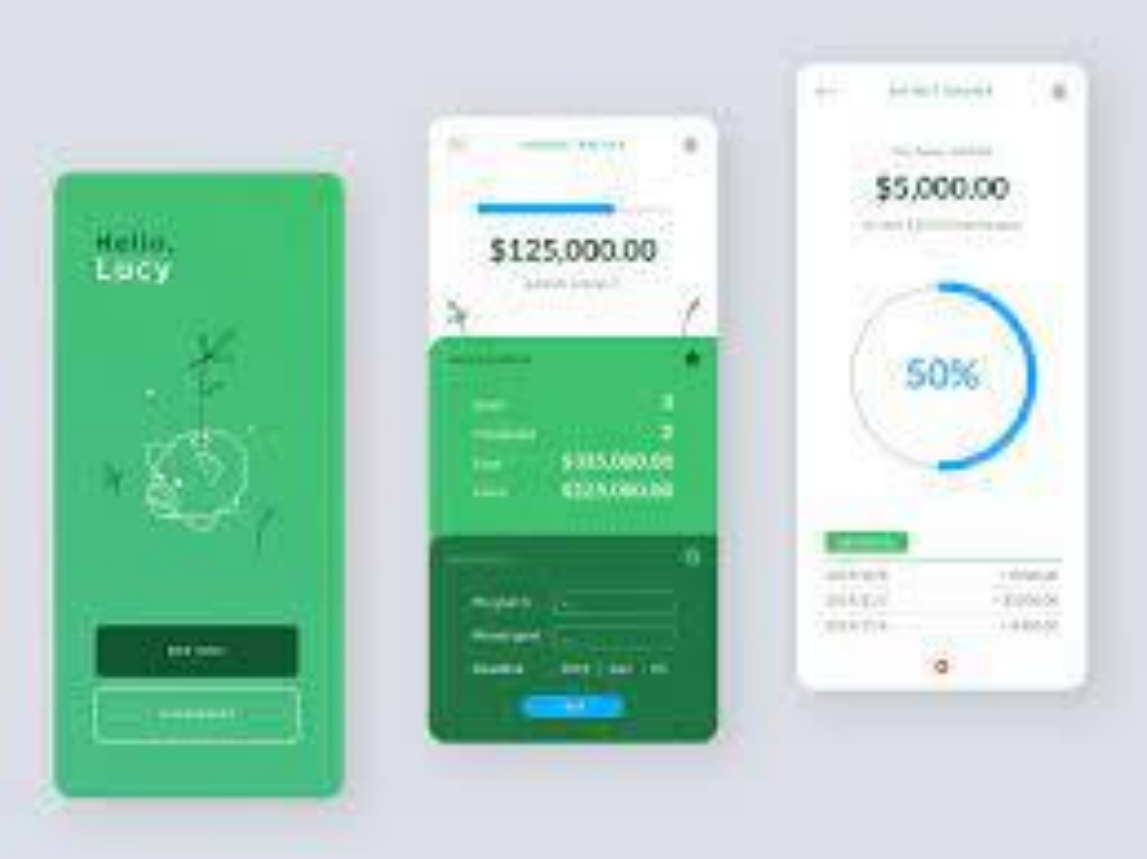

Category #3: Savings Tracker

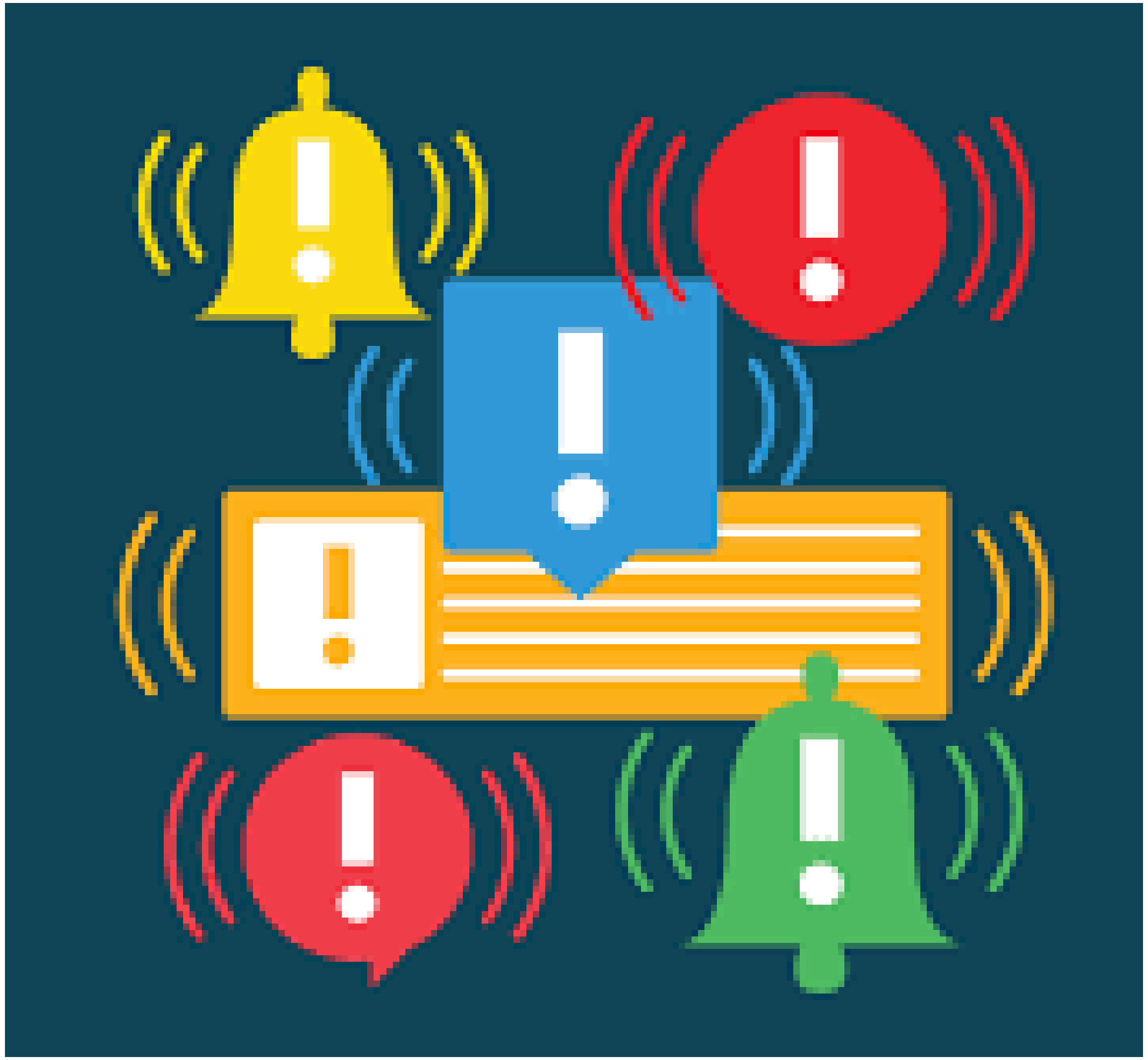

Category #4: Notifications

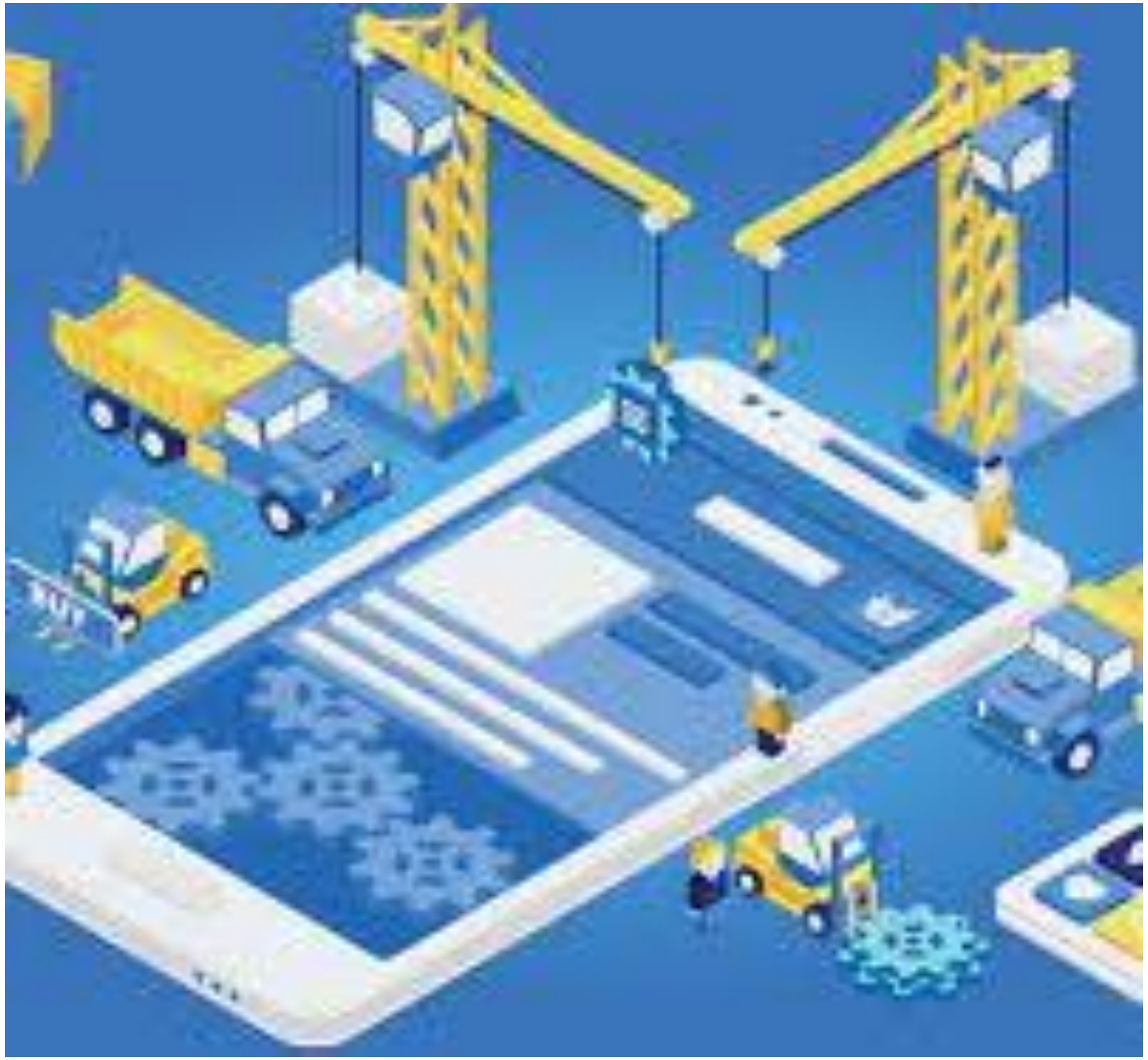

Category #5: Customization

Final Thoughts

What additional information would you like to see from a mobile app related to smoking cessation?

- More categories?

Have you used other apps to improve your health?

- Did you like using them? Why or why not?

Is there anything we missed?

Thank you for your participation!
